# Electrically Conductive Micropatterned Polyaniline-Poly(ethylene glycol) Composite Hydrogel

**DOI:** 10.3390/ma14020308

**Published:** 2021-01-08

**Authors:** Soyoung Noh, Hye Yeon Gong, Hyun Jong Lee, Won-Gun Koh

**Affiliations:** 1 Department of Chemical and Biomolecular Engineering, Yonsei University, 50 Yonsei-ro, Seodaemun-gu, Seoul 03722, Korea; ncyncy@naver.com (S.N.); hyk2049@gmail.com (H.Y.G.); 2 Department of Chemical and Biological Engineering, Gachon University, 1342 Seongnamdaero, Gyeonggi-do 13120, Korea

**Keywords:** electrically conductive hydrogel, hydrogel micropatterns, polyaniline, myogenic differentiation

## Abstract

Hydrogel substrate-based micropatterns can be adjusted using the pattern shape and size, affecting cell behaviors such as proliferation and differentiation under various cellular environment parameters. An electrically conductive hydrogel pattern system mimics the native muscle tissue environment. In this study, we incorporated polyaniline (PANi) in a poly(ethylene glycol) (PEG) hydrogel matrix through UV-induced photolithography with photomasks, and electrically conductive hydrogel micropatterns were generated within a few seconds. The electrical conductance of the PANi/PEG hydrogel was 30.5 ± 0.5 mS/cm. C2C12 myoblasts were cultured on the resulting substrate, and the cells adhered selectively to the PANi/PEG hydrogel regions. Myogenic differentiation of the C2C12 cells was induced, and the alignment of myotubes was consistent with the arrangement of the line pattern. The expression of myosin heavy chain on the line pattern showed potential as a substrate for myogenic cell functionalization.

## 1. Introduction

Hydrogel-based cellular patterns have been widely used in various biomedical applications, such as biosensors, tissue engineering, and cellular behavior observation [1,2,3,4]. The cell micropattern derived by modifying the hydrogel surface or geometry affects cell behaviors, such as proliferation and differentiation, according to the micropattern shapes and sizes [3,4,5]. A cellular pattern is a suitable tool for the efficient observation of cells in a small area, and various functions have been adapted to the microcellular pattern system. The endowment of an electrically conductive property to the hydrogel pattern is critical for applying neural or myogenic cells. Some researchers combined soft materials and electrically conductive property for stretchable bioelectronics [6,7,8], cardiac tissue engineering [9], and wound healing [10]. Nevertheless, few successful electrically conductive hydrogel micropatterns were synchronized with cell patterning because combining the hydrogel patterning with an electrically conductive material is challenging.

The electrically conductive hydrogel pattern system is a promising tool in the biomedical field, especially for myogenic cells. Muscle tissue consists of distinguishing characteristics, such as aligned myofiber of the myogenic cells, elasticity, and electrical conductance [11,12,13]. There are examples of electrically conductive patterning on silicon-based materials, where C2C12 cells were cultured on the substrates [14,15,16]. However, the substrate modulus of silicon-based materials is not appropriate for mimicking the natural cellular environment. In contrast, hydrogel-based materials provide an acceptable range of mechanical modulus for myogenic cells. There have been some achievements in micropatterning electrically conductive hydrogels and evaluating their biomedical applications [12,13]. Micro-grooves were generated on the electrically conductive hydrogel surface through laser ablation [12] or micro-molding [13], and electrical stimulation was applied to demonstrate the effect of electrical conductivity. One limitation of the previous studies was using only one electrical current to the entire substrate because all the electrically conductive parts were connected.

Moreover, myogenic cells require electrically conductive patterns and a muscle-like modulus to mimic the native tissue environment. The substrate modulus has a significant effect on cellular behavior, and it is being studied in a discipline called mechanotransduction [17,18]. Thus, the fabrication of cellular micropatterns on electrically conductive hydrogel materials is necessary for cells that require a soft substrate and electrical conductivity.

Polyaniline (PANi), a delocalized π-system backbone along the polymer chains, is one of the most widely used conductive polymers because of its low cost, wide availability, and excellent stability [19]. PANi can exist under three different oxidation states: the completely reduced state (leucoemeraldine base); the completely oxidized state (pernigraniline base); and the emeraldine base, consisting of repeating segments of alternating oxidized and reduced units in its structure [20,21,22]. Among these three types, the emeraldine salt form shows the highest electrical conductivity [21,22]. The polymerization of the aniline monomers facilitates the electrical conductance of hydrogel-based materials in the hydrogel matrix or at the interface between the organic solvent and hydrogel networks [23,24]. The in-situ polymerization of aniline provides uniform bulk properties. Still, this process requires a polymerization time of at least 4 h and a purification time of two days to remove residual aniline monomer.

In the present study, we attempted to fabricate, within a few seconds, a hydrogel-based substrate constructed with electrically conductive micropatterns using the photolithography of PANi with hydrogel. Crosslinked poly(ethylene glycol) (PEG) was used as the hydrogel matrix, and the PANi was incorporated into the hydrogel matrix via UV-induced photo-crosslinking following the blending of a PEG-diacrylate (PEG-DA) hydrogel precursor solution and electrically conductive PANi. During the photolithography process, the PANi/PEG hydrogel pattern was confined by photomasks, and micropatterns of electrically conductive hydrogels were generated through 7 s of UV radiation. The non-adhesive area for cells around the PANi patterns was simultaneously fabricated by a second UV-induced crosslinking of the PEG hydrogel substrate without PANi. Surface properties, such as morphology, water contact angle, and electrical conductance, were evaluated. The difference between our substrate and those of the previous studies was a disconnected pattern of the electrically conductive hydrogel material. Each pattern domain was independent, so different currents could be applied to each pattern domain simultaneously if needed. Although an electrical stimulus was not involved in this study, the material could be an electrical property-based multiplex cellular pattern system. To demonstrate the feasibility of the electrically conductive micropatterned hydrogel for application in myogenic cell culture systems, we cultured C2C12 myoblasts on patterned substrates. Cellular patterns, viability, alignment, and differentiation on the line-patterned, electrically conductive hydrogel were investigated.

## 2. Materials and Methods

### 2.1. Materials

Unless otherwise noted, all chemicals and solvents were of analytical grade and used as provided by the manufacturers. Polyaniline(emeraldine salt), (1R)-(-)-10-Camphorsulfonic acid 98% (CSA), poly (ethylene glycol) diacrylate (PEG-DA, MW 575 Da), N,N-dimethyl formamide (DMF), 2-hydroxy-2-methylpropiophenone (HOMPP), poly-l-lysine, ethanol, 0.01 M phosphate-buffered saline (PBS), bovine serum albumin (BSA), sulfuric acid, dimethyl sulfoxide (DMSO), 3-(4,5-dimethylthiazol-2-yl)-2,5-diphenyltetrazolium bromide (MTT), and Triton X-100 were purchased from Sigma-Aldrich (Milwaukee, WI, USA). Dulbecco’s modified Eagle medium/nutrient mixture F-12 (DMEM/F-12), fetal bovine serum (FBS), antibiotic/antimycotic solution, Dulbecco’s modified phosphate-buffered saline (DPBS), Neurobasal^®^ Media, 2% B-27^®^ supplement, Gibco^®^ GlutaMAX supplement, 1% penicillin-streptomycin, trypsin/ethylenediaminetetraacetate (trypsin/EDTA), calcein AM, Alexa Fluor 488, 594 Protein Labeling Kit, and 4′,6-diamidino-2-phenylindole (DAPI) were purchased from Thermo Fisher Scientific (Waltham, MA, USA). Alexa Fluor 488-labeled MYH antibody (sc-376157) was purchased from Santa Cruz Biotechnology, Inc. (Santa Cruz, CA, USA).

### 2.2. Fabrication of Micropatterned Conductive Hydrogel

A PANi solution was prepared by dissolving polyaniline (emeraldine salt) powder and CSA in DMF at 15 *w*/*v*% and placing the solution on a hotplate at 60 °C for 30 min with stirring. The PEG hydrogel precursor solution was prepared by dissolving 5 *v*/*v*% HOMPP and 50 *v*/*v*% PEG-DA in deionized (DI) water. The resultant PANi solution and PEG hydrogel precursor solution were mixed at a 1:1 volume ratio and placed on a hotplate at 60 °C for 1 h with stirring. The mixed solution was dropped onto a 2.4 × 2.4 cm^2^ glass slide, and a photomask was placed on top of the solution to form micropatterns with a uniform thickness. The glass slide was then exposed to UV light (365 nm, 300 mW/cm^2^, EFOS Ultracure 100ss Plus, Mississauga, ON, Canada) for 7 s. The photomask was detached after micropatterning with gelation of the PANi/PEG mixed solution. The resultant PANi/PEG micropatterns were gently washed once and immersed in DI water to remove residual PANi particles and precursor solution.

The PEG hydrogel precursor solution was applied onto the resultant conductive hydrogel pattern to make a free-standing substrate containing a micropatterned conductive hydrogel. A transparent photomask film without a pattern was placed on top to form a substrate of uniform thickness, and the material was exposed to UV light for 1 s through the transparent photomask film. The resultant hydrogel substrate, which contained the conductive micropattern, was detached from the glass slide, washed once with 95 *v*/*v*% ethanol, and rinsed with DI water.

The surface of the micropatterned conductive hydrogel substrate was coated using poly-l-lysine to provide a cell adhesion motif. The substrate was immersed in a 6 mg/mL poly-l-lysine solution at 37 °C for 30 min.

The resulting micropatterned conductive hydrogel was observed using an inverted fluorescence microscope (Olympus FV 1000, Tokyo, Japan) to obtain optical microscopic images and fluorescence images. The surface morphologies of the PANi/PEG and PEG hydrogels were observed by scanning electron microscopy (SEM; S-4700, Hitachi, Tokyo, Japan) at the Smart Materials Research Center for IoT at Gachon University. The average electrical conductivity of the fully swollen hydrogel was measured using a standard four-point probe technique (RT- 70/RG-5 four-point probe system, Napson, Tokyo, Japan) at room temperature.

### 2.3. C2C12 Myoblasts Culture on Micropatterned Conductive Hydrogel

Mouse C2C12 myoblasts were purchased from the American Type Culture Collection (ATCC, Rockville, MD, USA). The cells were cultured in tissue culture flasks using DMEM/F-12 containing 10 *v*/*v*% FBS and 1 *v*/*v*% penicillin-streptomycin, and the confluent cells in the flasks were detached by trypsinization. The cells (numbering 3.0 × 10^4^) were seeded onto the micropatterned conductive hydrogel substrate and incubated at 37 °C for 1 h without the addition of a culture media. The cell-adhered substrate was transferred to a new 24-well plate with gentle washing to remove non-adherent cells, and fresh culture media were supplied.

The C2C12 cells were stained for observation under a fluorescence microscope using calcein-A and incubated with 2 μM calcein-AM for 1 h at 37 °C. The MTT assay was used to measure the cytocompatibility of the cells. The cells on the micropatterned conductive hydrogel substrate were incubated with 10 *v*/*v*% MTT solution (6 mg/mL) in the culture media at 37 °C for 1 h. DMSO was added to dissolve the formazan crystals transformed from MTT, and the absorbance at 540 nm was measured using a microplate reader (SpectraMAX, Molecular Devices, Sunnyvale, CA, USA).

The C2C12 myoblasts were differentiated in differentiation media containing high DMEM, 2 *v*/*v*% horse serum, and 1 *v*/*v*% penicillin/streptomycin for 6 days after the C2C12 myoblasts were stabilized on the scaffold for 3 days in a growth media (high DMEM, 5 *v*/*v*% FBS, and 1 *v*/*v*% penicillin/streptomycin). The media were changed every 2 days during the cultural period. Differentiated C2C12 myotubes were fixed with a 4 *v*/*v*% paraformaldehyde solution for 20 min. Cell membranes were permeabilized by treatment with 0.2 *v*/*v*% Triton X-100 for 1 h, followed by 1 *w*/*v*% BSA solution for 1 h to block non-specific binding. The prepared samples were incubated overnight at 4 °C with 0.1 *v*/*v*% mice monoclonal antibody to the Alexa Fluor 488-labeled MYH, and the nuclei were stained with 0.1 *v*/*v*% DAPI for 5 min. DPBS washing steps were included in each step. The fluorescence images were obtained using a confocal microscope (LSM 700; Carl Zeiss, Oberkochen, Germany).

### 2.4. Statistical Analysis

All data are expressed as mean ± standard deviation (SD). Each experiment was repeated at least three times unless otherwise indicated. Statistical evaluation was performed by one-way ANOVA using the statistical software GraphPad Prism (Version 8.4). A value of *p* < 0.05 was considered statistically significant. 

## 3. Results

### 3.1. Fabrication of Micropatterned Conductive Hydrogel

**Figure 1:**
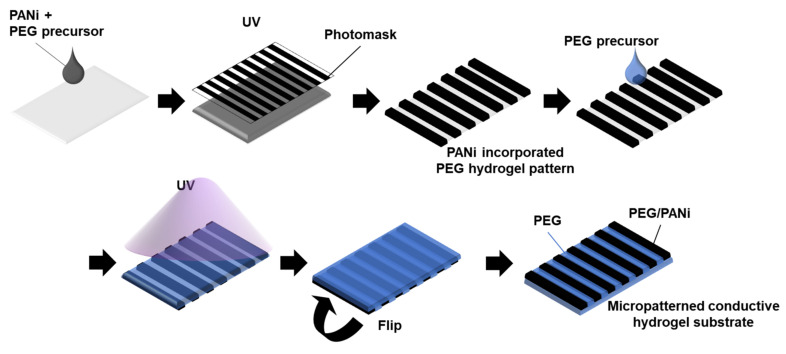
Schematic image of the fabrication of the micropatterned electrically conductive hydrogel.

PANi was incorporated into a PEG-based hydrogel substrate to introduce electrical conductivity. The process of using PEG-DA to pattern a PANi-incorporated PEG hydrogel was well established in previous studies [25]. In this study, PANi powder was mixed with the hydrogel precursor solution (Figure 1). The manufacturing process consisted of sequential hydrogel crosslinking using UV irradiation. The first UV irradiation was performed with a photomask to generate an electrically conductive micropattern of the PANi/PEG-DA mixture. The type of photomask could adjust the pattern shapes and sizes. The second UV irradiation was applied to the PEG-DA hydrogel precursor solution without the photomask to generate regions that prevented the attachment of biomolecules. In addition, the free-standing substrate was developed by filling in the gaps among the conductive patterns.

**Figure 2:**
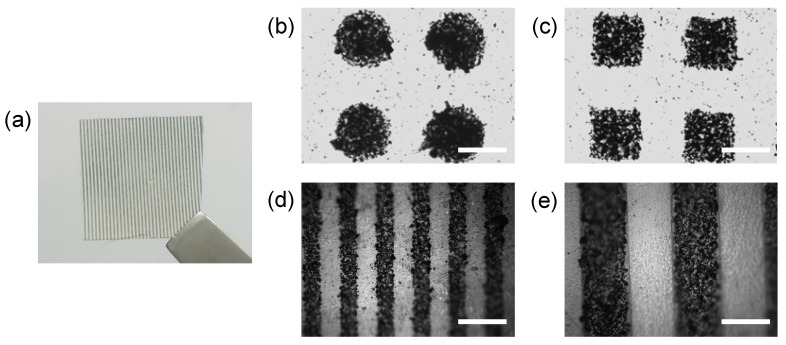
(**a**) Photographic image of a micropatterned conductive hydrogel. Optical microscope images of (**b**) circular and (**c**) rectangular micropatterns of the electrically conductive region, and line patterns with (**d**) 100 and (**e**) 200 μm of width and spacing. Scale bars: 200 μm.

The resulting material was a free-standing hydrogel thin film, including micropatterned conductive regions (Figure 2a). PANi-incorporated PEG (PANi/PEG) hydrogel regions exhibited a dark color due to the PANi powder, and the plain PEG hydrogel regions were transparent. The pattern shape, such as a circle or square, was adopted from the photomask (Figure 2b,c), and the width and spacing of the line patterns were also controllable (Figure 2d,e). Although some PANi were randomly scattered within the plain PEG hydrogel region during the two-step hydrogel curing process, PANi/PEG and plain PEG hydrogel regions and their boundary were distinguishable.

**Figure 3:**
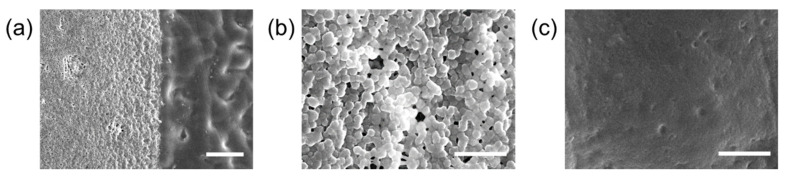
SEM images of the micropatterned conductive hydrogel. (**a**) The boundary between the PANi/PEG (**left**) and PEG (**right**) hydrogels. Scale bar: 20 μm; (**b**) the PANi/PEG region; and (**c**) the plain PEG region. Scale bars: 3 μm.

The boundary between the PANi/PEG and plain PEG hydrogels was identified by the difference in surface morphologies in the SEM image (Figure 3a). While the PEG hydrogel surface had a relatively smooth morphology, the PANi/PEG hydrogel region possessed a porous surface with the PANi. In addition, the PANi formed particle shape with approximately 200–300 nm in diameter and composed well-packed structures in the PEG hydrogel matrix (Figure 3b). In addition, some dark dots were observed in the plain PEG hydrogel region in the optical images (Figure 2), but no particles were exposed on the surface (Figure 3c).

**Figure 4:**
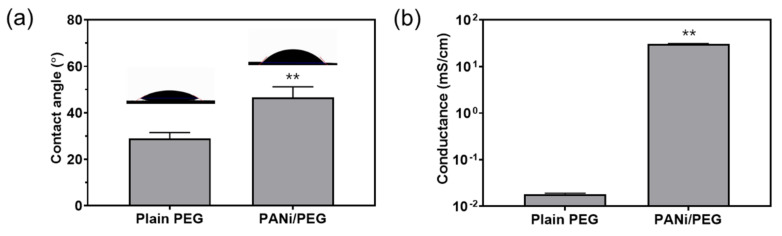
Physical properties of the plain PEG and PANi/PEG hydrogels: (**a**) contact angle and (**b**) electrical conductivity. Significant differences are represented by *p* < 0.01 (**).

The contact angles and electrical conductivities of the patterned PANi/PEG and plain PEG hydrogels were evaluated and compared. The contact angle of the plain PEG hydrogel was 29.0 ± 2.5°, while that of the PANi/PEG hydrogel was 46.6 ± 4.6° (Figure 4a), indicating that the incorporation of PANi in the PEG hydrogel matrix increased its hydrophobicity. In addition, the electrical conductivity of the PEG hydrogel matrix was significantly enhanced by the PANi. The electrical conductivities of the plain PEG and patterned PANi/PEG hydrogel were 1.83 ± 0.08 × 10^−3^ mS/cm and 30.5 ± 0.5 mS/cm, respectively (Figure 4b). The result represents an approximate 1667-fold increase in conductivity using the PANi particles obtained by a facile fabrication method.

### 3.2. Cellular Proliferation

**Figure 5:**
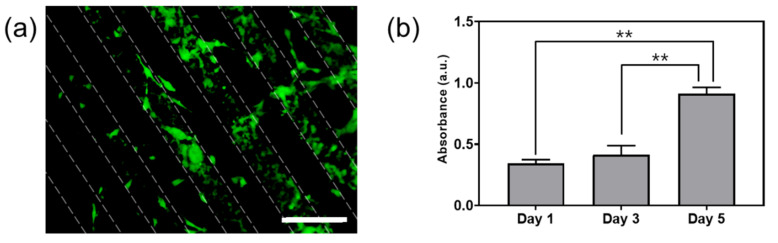
(**a**) Fluorescence image of C2C12 cells on the 100 μm line-micropatterned, conductive hydrogel. Scale bar: 200 μm; (**b**) cellular proliferation of C2C12 cells on the 100 μm line-micropatterned, conductive hydrogel. Significant differences are represented by *p* < 0.01 (**).

C2C12 cells, a myoblast cell line, were cultured to evaluate the effects of the morphology and conductivity of the hydrogel substrate. The cells were seeded on the micropatterned, conductive hydrogels containing line patterns 100 μm in width. Some cells adhered to the plain PEG hydrogel regions, but most cells attached to the conductive, patterned areas. Thus, a cellular micropattern could be produced along with the PANi-incorporated conductive pattern. The cytocompatibility of the PANi-incorporated conductive hydrogel was assessed through an MTT assay without differentiation with time (Figure 5b). On day 3, the absorbance of the MTT assay increased slightly compared to day 1. However, the absorbance effectively doubled between day 3 and day 5, which showed that this period was the most active for cell metabolism. The increase in the MTT assay level with time suggests that the conductive hydrogel provided a non-cytotoxic environment conducive to cellular growth.

### 3.3. C2C12 Cell Alignment and Differentiation

**Figure 6:**
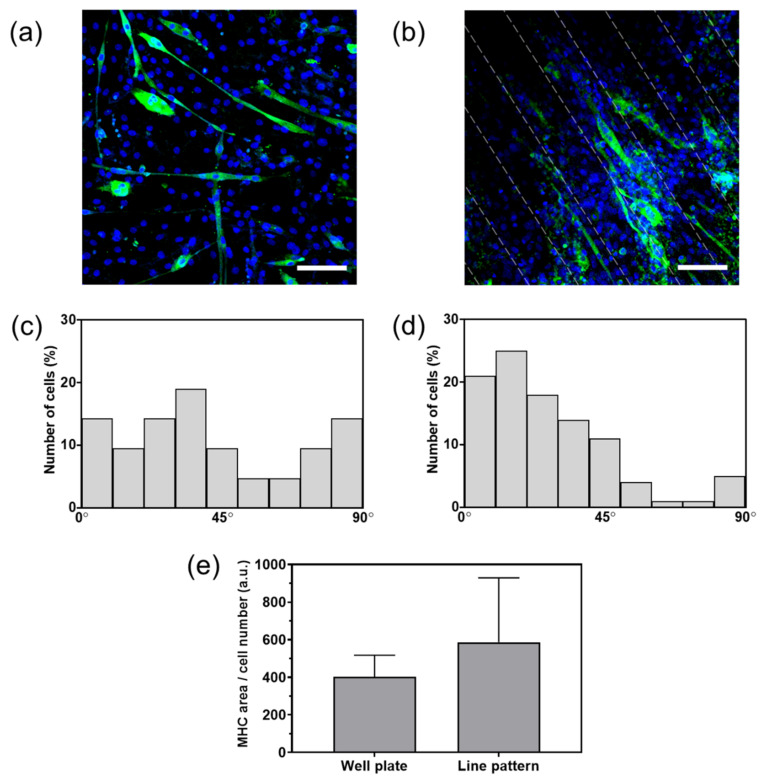
Immunofluorescence images of the nuclei (blue) of myosin heavy chain (MHC; green) of cells cultured on (**a**) a well plate and (**b**) 100 μm line-patterned PANi/PEG hydrogel. Scale bars: 100 μm. Myotube alignment of the C2C12 cells on (**c**) a well plate and (**d**) 100 μm line-patterned PANi/PEG hydrogel. Myotube alignment was measured by the angle between the long axis of the cells and the direction of the line pattern; (**e**) fluorescence area of the MHC per cell numbers. No significant difference.

The effect of the electrically conductive micropattern on the differentiation of the C2C12 cells was evaluated by myosin heavy chain (MHC) formation. The cells were cultured in differentiation media for six days, and the nuclei and MHC were then stained (Figure 6a,b). The cells cultivated on the well plate and the 100 μm line-patterned PANi/PEG hydrogel showed MHC formation with green fluorescence.

Additionally, the number of myotubes extending from the cells at a given angle between the myotube direction and the line pattern was counted (Figure 6c,d). The center horizon line was set to 0° relative to the myotube elongation angles to measure the number of myotubes on the well plate. The cells on the well plate showed a homogeneous distribution without bias toward a specific angle range. In contrast, approximately 65% of the cells cultured on the line-patterned hydrogel exhibited angles between 0–30°, which indicates that the myotubes tend to grow along with the conductive line pattern.

MHC expression was quantitatively analyzed by the fluorescence area of MHC per cell number (Figure 6e). There was no statistical difference, but the cells on the line pattern showed an MHC area approximately 1.5 times higher than that of the cells cultured on the well plate. The result indicates that the conductive micropattern increased the differentiation of the C2C12 cells.

## 4. Discussion

In the current study, PANi was incorporated into the PEG hydrogel matrix to introduce electrical conductivity. PANi emeraldine, a commercially available salt powder, was dissolved and blended with a PEG hydrogel precursor solution. A PANi/PEG hydrogel was fabricated using a conventional PEG-DA (PEG-diacrylate) photo-crosslinking method (Figure 1). Although the substrate modulus was not measured in this study, it can be easily controlled by the molecular weight of PEG-DA, according to the intended purpose. A previous study showed that Young’s modulus of a hydrogel fabricated with a PEG-DA molecular weight of 3.4 kDa was approximately 30 kPa, which was sufficient for myogenic cell culture [13].

Incorporating PANi in the hydrogel matrix is a time-effective way to fabricate conductive hydrogel patterns without polymerizing PANi. With the in-situ polymerization performed in previous research, interfacial polymerization was applied to fabricate the PANi/PEG hydrogel and salt leaching. The process required UV photocrosslinking to develop the PEG hydrogel, ammonium persulfate immersion, aniline monomer immersion, and PANi polymerization steps [24]. The entire process took approximately five days, including rinsing and removal of the residual monomers, while our proposed conductive hydrogel pattern fabrication process was completed within 10 min. Moreover, the absence of by-products and residue from the PANi synthesis reaction in our conductive hydrogel fabrication results in a non-cytotoxic product because PANi itself is biocompatible [26].

The resulting hydrogel substrate exhibited two distinct regions, the dark and transparent areas (Figure 2); the dark-colored area comprised the PANi, which are dark green. The thickness of the PANi/PEG structure could be controlled by adjusting the volume of the PANi/PEG-DA precursor solution before the first UV exposure. The total thickness of the substrate could be adjusted by the PEG-DA precursor solution, which constitutes the transparent region. The flexibility of the substrate can be adjusted by changing the thickness. Thin hydrogels are highly flexible and appropriate for tissue engineering, while thick hydrogels are robust and useful for substrates, such as biosensors.

The UV exposure time was also adjusted depending on the hydrogel material. Because the PANi exhibited a dark color, they hindered the penetration of the UV light. Thus, the UV exposure time for the PANi/PEG precursor solution mixture was 7 s, while the ordinary UV exposure time for the transparent PEG hydrogel precursor solution was 0.5–1 s. We confirmed that patterns 100 μm in scale were successfully fabricated with clear boundaries between the PANi/PEG and plain PEG regions (Figure 2). Generally, an increase in the UV exposure time decreased the resolution of the pattern size. A smaller line pattern was attempted using the same UV-induced crosslinking, resulting in uneven width and spacing. Thus, we fixed the line pattern size to 100 μm because it was the minimum resolution possible for this study. The surface geometry was measured by surface profiler, and the PANi-incorporated region showed relatively low height compared to the plain PEG hydrogel region (Figure S1). Due to the short exposure time of the second UV irradiation, the UV could not reach the bottom of the PANi-incorporated region by the opacity of PANi. As a result, after flipping the substrate, the geometry of the PANi/PEG composite part was lower and uneven compared to the PEG hydrogel region.

The transparency of the hydrogel precursor solution could be increased by reducing the amount of dark PANi, and the minimum resolution of the pattern size could be reduced by decreasing the concentration of PANi. However, the concentration of 7.5 *w*/*v*% PANi in the hydrogel precursor solution provided 30.5 ± 0.5 mS/cm of electrical conductivity, and it is appropriate for biomedical applications. The interconnectivity of the PANi particles is a crucial factor to endow the hydrogel with electrical conductivity, which would be significantly reduced by poor connections.

The surface properties of the hydrogel were dramatically changed by incorporating PANi. PANi was exposed at the surface, as shown in Figure 3a,b and enhanced the hydrophobicity of the surface. The water contact angle increased to 46.6 ± 4.6° from 29.0 ± 2.5° by blending the PANi with the PEG hydrogel matrix. This transition implies that the cells could adhere to the surface of the PANi-incorporated hydrogel. In contrast, the plain PEG hydrogel surface maintained a non-adhesive property, such that the cells attached selectively to the PANi/PEG hydrogel surface.

In most cases, cell-binding proteins are required for cell adhesion; therefore, the efficiency of cell adhesion is related to the ability of proteins to adsorb to the surface. Generally, a contact angle of 40°–100° exhibited a cell adhesion efficiency of over 60%, which is adequate for cells to adhere to the surface [27]. Thus, the PANi/PEG hydrogel surface, which showed a contact angle of about 47°, would be more suitable for cell adhesion than the plain PEG hydrogel surface, which showed a contact angle of about 29°.

The surface conductivity of the PANi/PEG hydrogel was measured by the four-point probe method. PANi incorporation increased the electrical conductivity approximately 1667-fold to 30.5 ± 0.5 mS/cm (Figure 4b). Although the conductivity was about 1000–5000 times lower than that of bulk PANi (3000–20,000 mS/cm) [19], it is sufficient for biomedical applications. Native muscle tissues exhibit electrical conductivity in the range of 1–10 mS/cm [11,13], the electrical conductivity of approximately 30 mS/cm is appropriate for myogenic cellular applications.

C2C12 cells were cultured on the micropatterned conductive hydrogel for the evaluation of the topographical and electrical properties. C2C12 is a cell line of myoblasts, and the cells have been cultivated with micropatterns and electrical stimulation [3,4,12,13,28,29,30]. Previous studies showed that myogenic differentiation and alignment of C2C12 cells were most enhanced by line pattern distances in the range of 50–100 μm [3,4,12]. In the current study, we selected 100 μm line pattern distances for the cell experiments. As mentioned, the resolution of the photolithography technique was limited to lengths of 100 μm, so we chose the minimum resolution condition that could be fabricated successfully.

As expected from the contact angle results, the cells tended to adhere more to the PANi/PEG regions than the plain PEG hydrogel surfaces because the PANi/PEG surfaces were more favorable for lysine adsorption and cell adhesion (Figure 5a). The result indicates that the fabricated PANi/PEG hydrogel pattern provided a pattern of conductive regions and cellular patterns for cell adhesion. The distribution of cell adhesion varied from line to line, and the cell optimal cell density would be determined depending on the width and space of line patterns. When the cells were cultured on the circular pattern, the cell distribution was relatively even compared to the line pattern (Figure S2). The cell adhesion and distribution could differ depending on the pattern shapes and sizes. We will observe the effect of the pattern on the cells in the next study. Cell viability was evaluated by MTT assay, and the absorbance increased with culture time (Figure 5b). The cells grew on the substrate without a decrease in the total cell number, so the PANi powder-incorporated conductive hydrogel substrate provided a cytocompatible environment for cell cultures.

The effects of the micropattern and electrical conductivity on myogenic differentiation were evaluated by immunostaining for MHC and myotube alignment. MHC expression represents microtubular formation, which is a widely used myogenic differentiation marker [12,13]. C2C12 cells were cultured on a well plate and on a 100 µm line-patterned, electrically conductive hydrogel in the differentiation media to evaluate the myogenic differentiation of the C2C12 cells. The cells on the electrically conductive micropatterned hydrogel were aligned along with the line patterns, while there was no tendency toward cell orientation on the well plate (Figure 6a–d). Three parameters were differentiating the well plate and the 100 µm line-patterned, conductive hydrogel: the substrate modulus, micropattern, and electrical conductivity. Among them, we predict that the micropattern promoted cellular alignment. Park et al. showed that myotubes were highly elongated along the direction of the line pattern while they were stretched in random directions on the non-patterned substrate; thus, even in the presence of electrical conductivity, cellular alignment, or stretching was not promoted without micropatterns [12]. Our substrate demonstrated that cellular alignment could be achieved through micropatterns generated by the photolithography process.

In addition, the MHC area per cell number was quantified via image analysis. The green region, which represented MHC expression, was divided by the number of blue dots, representing cell nuclei (Figure 6e). The results showed that the line-patterned, conductive hydrogel substrate slightly increased myogenic differentiation, although there was no statistical difference compared to the myogenic differentiation of C2C12 on the well plate. The electrical conductivity of the substrate could affect myogenic differentiation, but electrical stimulation is necessary to improve this effect [12]. Our cell culture system was not configured with an electrical stimulation system in the current study, which introduced some limitations in observing a noticeable improvement in the myogenic differentiation. Consequently, future studies using the combination of our micropatterned electroconductive hydrogel substrate and an electrical stimulation system will be necessary to demonstrate the highly effective myogenic differentiation.

## 5. Conclusions

We fabricated an electrically conductive micropattern on a hydrogel-based material via a rapid, facile two-step photolithography process. The surface morphology and properties were evaluated, and the electrical conductivity was found to be adequate for cellular applications. The cellular pattern was created on a PEG hydrogel matrix with an electroconductive PANi pattern, and the resulting hydrogel scaffold was cytocompatible. In addition, the myogenic differentiation of C2C12 cells on the line-patterned, electrically conductive hydrogel demonstrated the feasibility of the substrate to facilitate myogenic cell differentiation. In this study, we focused on the simple fabrication process of the conductive hydrogel patterns, so we did not evaluate the effect of the cellular pattern itself. The control of the conductive pattern parameters, such as the shape and size, would be a significant factor in optimizing cellular proliferation and differentiation. We plan to assess the effect of the electrically conductive pattern using a PANi-incorporated, patterned PEG hydrogel substrate in a future study. The fabricated platform is appropriate for evaluating the impacts of the electrically conductive pattern of the hydrogel. Our proposed method has great potential for tissue engineering applications, biosensors, and cellular behavior studies due to the straightforward production process.

## Data Availability

The data presented in this study are available on request from the corresponding author. At the time the project was carried out, there was no obligation to make the data publicly available.

## Supplementary Materials

The following are available online at https://www.mdpi.com/1996-1944/14/2/308/s1, Figure S1: Graph line patterned substrate measured by surface profiler. The width and space of line patterns are 100 μm., Figure S2: Fluorescence image of C2C12 cells on the 200 μm circle-micropatterned, conductive hydrogel. Scale bars: 200 μm.
