# Supplementary material for: Electrically Conductive Micropatterned Polyaniline-Poly(ethylene glycol) Composite Hydrogel
Source: Materials (Basel). 2021 Jan 8;14(2):308. doi: 10.3390/ma14020308 (PMC7827658; doi:10.3390/ma14020308)
Supplement: Supplementary file 1 [file materials-14-00308-s001.zip › materials-1047802-supplementary.pdf]

materials
MDPI

Supplementary Materials

Electrically conductive micropatterned polyaniline-poly(ethylene glycol) composite hydrogel

Soyoung Noh, Hye Yeon Gong, Hyun Jong Lee and Won-Gun Koh

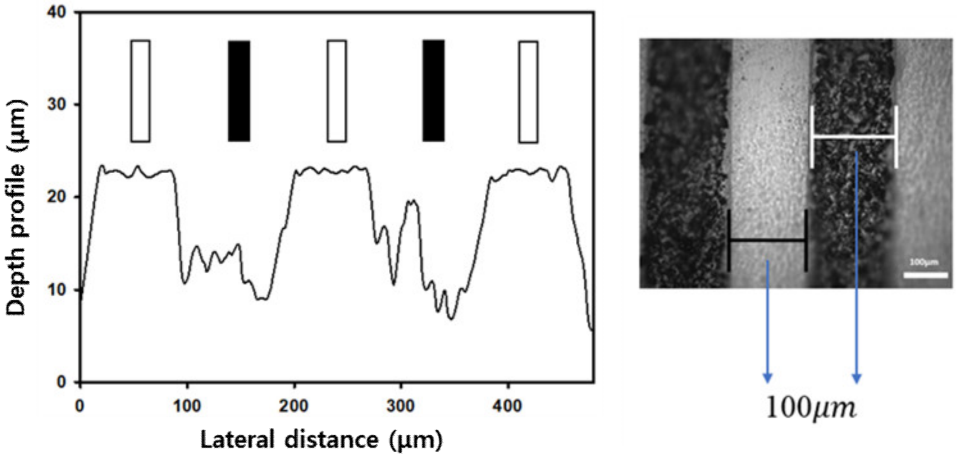

Figure S1. Graph line patterned substrate measured by surface profiler. The width and space of line patterns are 100 µm.

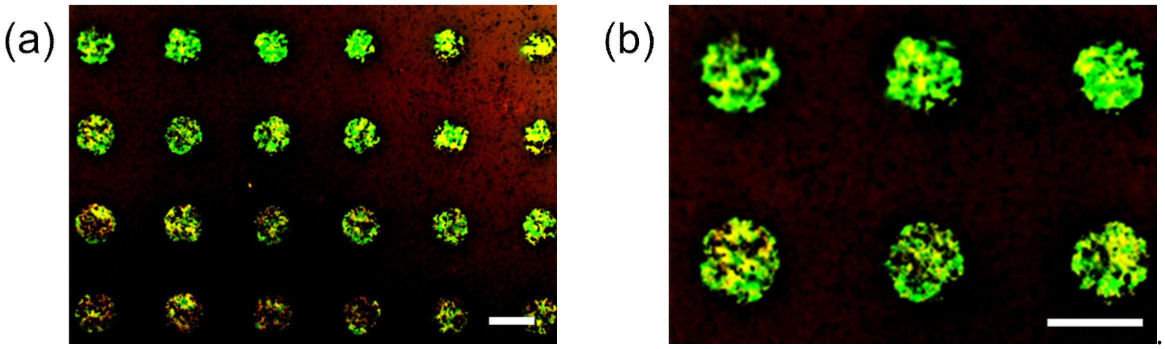

Figure S2. Fluorescence image of C2C12 cells on the 200 µm circle-micropatterned, conductive hydrogel. Scale bars: 200 µm.
